# Exploring Screen Time and Its Effects on Children’s Mental Health: A Cross-Sectional Study

**DOI:** 10.7759/cureus.71215

**Published:** 2024-10-10

**Authors:** Walaa Mulla, Wadeea Ahmed, Maryam Radhi, Huda Alaali, Ghufran Alwazeer, Fatema Yusuf, Ghaida Alsuhim, Atheer Al Suhaym, Walaa Alahmari, Mohamed Abdulla, Eman Yusuf

**Affiliations:** 1 Psychiatry, Psychiatry Hospital, Manama, BHR; 2 General Practice, Al Ghareeb Medical Center, Riffa, BHR; 3 College of Medicine, National University of Science and Technology, Muscat, OMN; 4 General Practice, Dr Suhair Al Saad Medical Centre, Sanad, BHR; 5 Medical Laboratories, Salmaniya Medical Complex, Manama, BHR; 6 College of Medicine, King Khalid University, Abha, SAU; 7 College of Medicine, Zhejiang University, Zhejiang, CHN

**Keywords:** bahrain, children's depression inventory, children's mental health, conduct problems, cross-sectional study, depressive symptoms, emotional symptoms, saudi arabia, screen time, strengths and difficulties questionnaire

## Abstract

Background

The increasing prevalence of screen-based activities among children has raised concerns about potential mental health effects. This study investigates the relationship between screen time and mental health outcomes in children aged 6 to 14 years in Saudi Arabia and Bahrain, focusing on emotional symptoms, conduct problems, hyperactivity, peer problems, and depressive symptoms.

Methods

A cross-sectional study was conducted with 670 children recruited through stratified random sampling. Data were collected using self-reported questionnaires and parental surveys from January to June 2024. Screen time was assessed across four activities: TV viewing, smartphone usage, computer/tablet use, and video game playing, recorded separately for weekdays and weekends. Mental health was evaluated using the Strengths and Difficulties Questionnaire (SDQ) and the Children’s Depression Inventory (CDI). Descriptive statistics and Pearson correlation coefficients were used to analyze the data.

Results

Participants reported a mean total screen time of 7.25 ± 2.4 hours on weekdays and 8.4 ± 2.65 hours on weekends. Smartphone usage (2.75 ± 1.3 hours/day) was the most common activity. Emotional symptoms, conduct problems and depressive symptoms were significantly correlated with both weekday and weekend total screen time (weekday r = 0.43, r = 0.31, and r = 0.49, respectively, p < 0.001; weekend r = 0.47, r = 0.33, and r = 0.54, respectively, p < 0.001). Smartphone usage had the strongest association with depressive symptoms (r = 0.46, p < 0.001).

Conclusion

This study identifies a significant positive correlation between increased screen time and adverse mental health outcomes in children, particularly emotional symptoms and depressive symptoms. The findings emphasize the need for public health strategies to manage screen time and promote healthier digital habits to mitigate the potential negative effects on children's mental health.

## Introduction

In recent years, the prevalence of screen-based activities among children has increased significantly, driven by the proliferation of digital devices such as smartphones, tablets, and computers. This rise in screen time has raised concerns about its potential impact on children’s mental health. Given the pivotal role of mental well-being in overall development, understanding how screen time affects mental health outcomes is crucial for developing effective intervention strategies [1-5].

Research suggests that excessive screen time is associated with various adverse mental health outcomes, including increased risks of anxiety, depression, and behavioral issues. Screen-based activities can displace physical activity and face-to-face social interactions, both of which are important for healthy psychological development. Additionally, the content and nature of screen exposure - such as violent media or cyberbullying - can further exacerbate these risks [2,6].

Mental health issues in children can manifest in various forms, including emotional symptoms, conduct problems, hyperactivity, and difficulties in peer relationships. Tools like the Strengths and Difficulties Questionnaire (SDQ) provide a comprehensive assessment of these behavioral and emotional difficulties. Depression, a particularly concerning issue, can severely impact a child’s quality of life and academic performance. The Children’s Depression Inventory (CDI) is a well-established instrument used to assess the severity of depressive symptoms in children [7-13].

The impact of screen time on mental health may vary across different cultural and regional contexts. Saudi Arabia and Bahrain, with their rapidly evolving digital landscapes and high levels of screen media consumption, present a unique setting for studying these effects. Understanding the specific influences of screen time on children’s mental health in these countries is essential for tailoring public health recommendations and interventions.

This study seeks to address the gap in research concerning the relationship between screen time and mental health outcomes in children within Saudi Arabia and Bahrain. By employing validated assessment tools and comprehensive data collection methods, the study aims to provide robust evidence on how screen time impacts various aspects of mental health. The findings will contribute to a better understanding of the effects of screen time and inform strategies for promoting healthier digital habits among children.

## Materials and methods

Study design

This cross-sectional study aimed to examine the impact of screen time on children's mental health in Saudi Arabia and Bahrain. The study adhered to ethical guidelines, and approval was obtained from the institutional review boards of the participating institutions.

Participants

Children aged 6 to 14 years were recruited for the study. Participants were selected through stratified random sampling to ensure a representative sample across various age groups and genders. Inclusion criteria required children to be within the specified age range, while those with known neurological or psychiatric disorders were excluded. Informed consent was obtained from parents or guardians, and children provided verbal assent.

Data collection procedures

Data were collected between January and June 2024. Recruitment occurred at schools, community centers, and healthcare facilities. Participants completed self-report questionnaires, and parents or guardians provided additional information through surveys. Data collection was conducted in a controlled environment to minimize distractions and ensure accurate responses. Data collectors were trained to administer the questionnaires consistently.

Measures

Screen time was assessed using a validated self-report questionnaire. Participants reported their average daily screen time across four activities: TV viewing, smartphone usage, computer/tablet use, and video game playing. Screen time was recorded separately for weekdays and weekends.

Mental health outcomes were assessed using the Strengths and Difficulties Questionnaire (SDQ) and the Children’s Depression Inventory (CDI). The SDQ evaluates behavioral and emotional difficulties through five subscales: Emotional Symptoms, Conduct Problems, Hyperactivity, Peer Problems, and Prosocial Behavior. Each subscale consists of five items rated on a 3-point scale (not true, somewhat true, certainly true). Higher scores in Emotional Symptoms, Conduct Problems, Hyperactivity, and Peer Problems indicate more significant difficulties, while higher scores in Prosocial Behavior reflect better functioning. The CDI assesses depressive symptoms with 27 items rated on a 3-point scale (0 = not at all, 1 = somewhat, 2 = very much). The total score ranges from 0 to 54, with higher scores indicating greater depressive symptoms. The CDI categorizes depression severity into Minimal (0-9), Mild (10-18), and Moderate to Severe (19-27).

Descriptive statistics were computed for demographic characteristics, screen time habits, and mental health scores. Continuous variables were summarized using means and standard deviations, while categorical variables were expressed as frequencies and percentages. Pearson correlation coefficients were calculated to assess the relationships between total screen time and mental health outcomes, analyzed separately for weekdays and weekends. Statistical significance was set at p < 0.05. Data analysis was performed using SPSS version 27 (IBM Corp., Armonk, NY, USA).

## Results

Demographics of study participants

A total of 670 participants were included in the study, with 340 from Saudi Arabia (50.7%) and 330 from Bahrain (49.3%). The mean age of participants was 10.4 years (± 2.2 years). The sample was evenly distributed by gender, with 351 (52.4%) males and 319 (47.6%) females. Regarding parental education levels, 59 (8.8%) had no formal education, 166 (24.8%) completed primary school, 282 (42.1%) had secondary school education, 136 (20.3%) attended college or university, and 27 (4.0%) had postgraduate education (Table 1).

**Table 1:** Demographics of study participants (N = 670) Percentages are based on the total number of participants (N = 670).

Characteristic		n (%)
Age (mean ± SD)		10.4 ± 2.2 years
Gender	Male	351 (52.4%)
	Female	319 (47.6%)
Parental Education Level	No formal education	59 (8.8%)
	Primary school	166 (24.8%)
	Secondary school	282 (42.1%)
	College/University	136 (20.3%)
	Postgraduate	27 (4.0%)

Screen time habits

Participants reported an average of 1.8 hours per day of TV viewing, 2.75 hours on smartphones, 1.4 hours on computers or tablets, and 1.45 hours playing video games. The total average screen time on weekdays was 7.25 hours per day, while on weekends, it increased to 8.4 hours per day. The most common types of content engaged included entertainment (61.3%, or 411 participants), social media (44.2%, or 296 participants), gaming (49.7%, or 333 participants), and educational programs (21.6%, or 145 participants) (Table 2).

**Table 2:** Screen time habits (N = 670) Screen time is reported as mean ± SD for each activity. Percentages for content engaged with are based on the total number of participants (N = 670).

Screen time activity		Mean ± SD (hours/day)
TV		1.8 ± 1.05
Smartphone		2.75 ± 1.3
Computer/Tablet		1.4 ± 0.85
Video Games		1.45 ± 1.15
Total Screen Time (Weekdays)		7.25 ± 2.4
Total Screen Time (Weekends)		8.4 ± 2.65
Primary Content Engaged With	Educational Programs	145 (21.6%)
	Entertainment	411 (61.3%)
	Social Media	296 (44.2%)
	Gaming	333 (49.7%)

Mental health assessment scores

The mean scores for the Strengths and Difficulties Questionnaire (SDQ) were as follows: Emotional Symptoms 4.2 (± 2.05), Conduct Problems 3.3 (± 1.65), Hyperactivity 4.95 (± 2.25), Peer Problems 3.45 (± 1.85), and Prosocial Behavior 6.85 (± 2.15). The Children’s Depression Inventory (CDI) total score averaged 14.0 (± 7.5). In terms of depression severity, 37.9% (254 participants) were classified with minimal depression, 44.5% (298 participants) with mild depression, and 17.6% (118 participants) with moderate to severe depression (Table 3).

**Table 3:** Mental health assessment scores (N = 670) Categorical data are represented as frequency and percentages, while continuous data are represented as mean and standard deviation. Percentages for categories are based on the total number of participants (N = 670).

Assessment Tool		Mean ± SD or n (%)
Strengths and Difficulties Questionnaire (SDQ)	Emotional Symptoms	4.2 ± 2.05
	Conduct Problems	3.3 ± 1.65
	Hyperactivity	4.95 ± 2.25
	Peer Problems	3.45 ± 1.85
	Prosocial Behavior	6.85 ± 2.15
Children’s Depression Inventory (CDI)	CDI Total Score	14.0 ± 7.5
	Minimal Depression (0-9)	254 (37.9%)
	Mild Depression (10-18)	298 (44.5%)
	Moderate to Severe Depression (19-27)	118 (17.6%)

Correlation between screen time and mental health scores

Correlation analyses revealed significant associations between screen time and mental health outcomes. Total screen time on weekdays was positively correlated with emotional symptoms (r = 0.43, p < 0.001), conduct problems (r = 0.31, p < 0.001), and CDI total score (r = 0.49, p < 0.001). Total screen time on weekends showed stronger correlations with emotional symptoms (r = 0.47, p < 0.001), conduct problems (r = 0.33, p < 0.001), and CDI total score (r = 0.54, p < 0.001). Smartphone usage also demonstrated significant correlations with emotional symptoms (r = 0.41, p < 0.001), conduct problems (r = 0.29, p < 0.001), and CDI total score (r = 0.46, p < 0.001). TV viewing and video game playing showed more moderate correlations with emotional symptoms and CDI total scores, while video game playing had a notable correlation with conduct problems (r = 0.33, p < 0.001) (Table 4).

**Table 4:** Correlation between screen time and mental health scores (N = 670) Correlation coefficients (r) are provided with p-values for significance testing. Percentages are based on the total number of participants (N = 670). P value is significant if <0.05.

Variable	Emotional Symptoms (SDQ)	Conduct Problems (SDQ)	CDI Total Score
Total Screen Time (Weekdays)	r = 0.43, p < 0.001	r = 0.31, p < 0.001	r = 0.49, p < 0.001
Total Screen Time (Weekends)	r = 0.47, p < 0.001	r = 0.33, p < 0.001	r = 0.54, p < 0.001
Smartphone Usage	r = 0.41, p < 0.001	r = 0.29, p < 0.001	r = 0.46, p < 0.001
TV Viewing	r = 0.25, p = 0.001	r = 0.21, p = 0.002	r = 0.28, p < 0.001
Video Game Playing	r = 0.37, p < 0.001	r = 0.33, p < 0.001	r = 0.41, p < 0.001

## Discussion

This study investigated the relationship between screen time and children’s mental health in Saudi Arabia and Bahrain using validated assessment tools. The results revealed significant associations between increased screen time and various mental health outcomes, including emotional symptoms, conduct problems, and depressive symptoms.

Our findings indicate that higher total screen time is positively correlated with emotional symptoms, conduct problems and depressive symptoms. Specifically, both weekday and weekend screen time showed strong associations with these outcomes. These results align with previous research suggesting that excessive screen time can negatively impact children’s mental health. The correlations observed were particularly pronounced for smartphone usage and total screen time on weekends, highlighting the potential influence of prolonged and intensive screen use on mental health [10-14].

Different types of screen-based activities exhibited varying degrees of association with mental health outcomes. Smartphone usage, which often includes social media and gaming, was strongly correlated with emotional symptoms and depressive symptoms. This finding supports the notion that the content and nature of screen interactions may play a critical role in influencing mental health. TV viewing and video game playing, while also associated with mental health outcomes, showed more moderate correlations compared to smartphone usage.

The CDI results revealed that a notable portion of participants experienced mild to moderate to severe depressive symptoms. This underscores the significant impact of screen time on depressive symptoms and highlights the need for targeted interventions. The observed relationship between increased screen time and higher CDI scores suggests that screen time may be a contributing factor to the development or exacerbation of depressive symptoms.

The findings have important implications for public health and policy. They underscore the need for guidelines and recommendations to manage screen time effectively. Health professionals and educators should emphasize the importance of balancing screen time with physical activity and face-to-face social interactions. Additionally, there is a need for parental education on the potential risks of excessive screen time and strategies to mitigate these risks [12-15].

Limitations

Despite the careful design of this study, there are some limitations to consider. The cross-sectional nature of the study limits our ability to draw causal conclusions about the relationship between screen time and mental health outcomes. Additionally, self-reported measures of screen time and mental health may be subject to biases, such as social desirability or recall bias. Future research should employ longitudinal designs and incorporate objective measures of screen time to provide a more comprehensive understanding of these relationships.

## Conclusions

This study reveals a significant relationship between increased screen time and adverse mental health outcomes in children, including emotional symptoms, conduct problems, and depressive symptoms. The results indicate that excessive screen use, particularly through smartphones and on weekends, is associated with a higher prevalence of mental health issues. These findings highlight the need for public health initiatives and parental guidance to manage screen time effectively and promote balanced lifestyles. By addressing these concerns, we can mitigate the potential negative impacts of screen time on children's mental well-being and support healthier developmental outcomes.
